# A novel dual-dimensional contrastive self-supervised learning-based framework for rolling bearing remaining useful life prediction

**DOI:** 10.1038/s41598-026-38417-7

**Published:** 2026-03-13

**Authors:** Zhunan Shen, Chenhao Yang, Liu Cheng, Xiangwei Kong, Zhitong Liu, Kaiyu Su

**Affiliations:** 1 School of Mechanical Engineering and Automation, Northeastern University, Shenyang, 110819 Liaoning China; 2 Key Laboratory of Vibration and Control of Aero-Propulsion System, Ministry of Education, Northeastern University, Shenyang, 110819 Liaoning China; 3 Liaoning Province Key Laboratory of Multidisciplinary Design Optimization of Complex Equipment, Northeastern University, Shenyang, 110819 Liaoning China

**Keywords:** Remaining useful life prediction, Rolling bearings, Self-supervised learning, Contrastive learning, Representation learning, Engineering, Mathematics and computing

## Abstract

Accurate bearing remaining useful life (RUL) can effectively ensure the safe operation of equipment and enhance production efficiency. Despite the widespread application of deep learning-based prediction methods, most rely on supervised learning to directly map input signals to output RUL. However, this often ignores crucial representational properties like smoothness and monotonicity, leading to disorganized and uninterpretable representations that significantly degrade performance. To enhance representation, this paper proposes a novel dual-dimensional contrastive self-supervised learning-based framework named DCSSL for RUL prediction of rolling bearings. It is carried out in two consecutive stages. In the first stage, a strategy combining random cropping and timestamp masking for constructing positive pairs for contrastive learning is proposed. The dual-dimensional contrastive loss function that combines temporal-level and instance-level is devised to enable the model to learn state representations in unlabeled vibration data and mine rolling bearing degradation trends. Then, in the second stage, RUL prediction of labeled vibration data is achieved by fine-tuning the newly constructed prediction head. Experimental validation of DCSSL on a large number of RUL prediction tasks demonstrates its superior performance over other state-of-the-art methods.

## Introduction

To effectively evaluate and enhance the life and performance of intelligent manufacturing equipment, prognostics and health management (PHM), which integrates multidisciplinary technologies, plays a pivotal role in fields such as mechanical engineering, railroad transportation, and metallurgical engineering. As a critical component of PHM, accurate Remaining Useful Life (RUL) prediction provides a scientific basis for maintaining rotating machinery such as rolling bearings. This capability not only informs timely maintenance actions but ultimately enhances both equipment safety and production economics^1–4^.

It is widely recognized that RUL prediction methods can be classified into physical modeling methods, data statistics methods, and deep learning methods. Physical modeling methods such as Paris-Erdogan^5^ require clear failure mechanisms and rich a priori knowledge, and statistical data methods such as Weibull distribution^6^ are heavily dependent on a substantial amount of failure data, both of which are forced to fade out of the historical stage due to their inherent limitations. The capabilities of deep learning have made these methods increasingly attractive for RUL prediction^7–11^. They not only reduce reliance on specialized expertise^12,13^ but also excel at extracting nonlinear, high-dimensional representations from data, thereby facilitating an end-to-end mapping from raw inputs to RUL predictions.

However, existing methods still face significant challenges: First, backbone networks such as Convolutional Neural Networks (CNNs), Graph Neural Networks (GNNs), Long Short-Term Memory networks (LSTMs), and Transformers are foundational to current methodologies, they are inherently limited by several critical constraints. These include restricted receptive fields, insufficient modeling of causal relationships, limited parallelization capability, over-reliance on specific topological assumptions, and high computational complexity, which collectively hinder their performance in complex RUL prediction scenarios. These constraints prevent them from effectively capturing long-term time-series dependencies during device degradation. Second, most end-to-end life prediction methods lack effective constraints on the representation, resulting in representations that are un-smooth, trendless, and lacking interpretability, yet these qualities are crucial for life prediction. Third, while recent approaches have begun exploring representation constraints, they predominantly follow typical self-supervised learning (SSL) paradigms, particularly self-supervised contrastive learning, which constructs sample pairs only at the instance-level. Although these methods can distinguish the health status of the devices, they are not sufficient to fully explore the various degradation manifestations embedded within the data and represent them in a smooth and continuous form in the feature space. Therefore, it is unable to depict the complete degradation trajectory^14^, which affects the accuracy of the prediction task.

To address the above-mentioned challenges, this paper proposes a novel dual-dimensional contrastive SSL-based framework (DCSSL). First, DCSSL employs the dilated causal convolution as its backbone network, which not only exponentially expands the receptive field but also ensures, through forward padding, that the representations extracted by the model are solely related to information preceding their corresponding time points. This enables the capture of long-term dependencies with minimal layers. Furthermore, its propagation mechanism aligns with standard CNN, resulting in efficient and stable training. Second, DCSSL imposes unique constraints on representations. Specifically, it performs contrastive learning across both instance and temporal levels, enabling the model to infer masked content based on context. This approach allows the model to understand the dynamic evolution patterns of vibration signals, ultimately developing the complete and smooth degradation trajectory of the equipment.

The main contributions of this study are summarized as follows:

1. A novel dual-dimensional contrastive SSL-based framework for RUL prediction of rolling bearings is proposed. It consists of two stages. In the first stage, smoothness and trend-based representations are extracted from unlabeled vibration data. In the second stage, RUL predictions for labeled vibration data are achieved by fine-tuning the newly constructed prediction head.
2. A dilated causal convolution backbone network is introduced. By leveraging the dilation mechanism, it can efficiently capture local representations. Moreover, due to its strict temporal causal principle, the model is capable of simultaneously capturing long-term sequence dependencies.
3. Conducting contrastive learning at both instance and temporal levels, where positive pairs are constructed through random cropping and timestamp masking. This enables the model to learn effective and interpretable representations from unlabeled vibration data and extract degradation trends in rolling bearings, significantly enhancing the model generalization capability.
4. The effectiveness of DCSSL is verified through a large number of experiments. Comprehensive ablation studies validate its exceptional performance across multiple evaluation dimensions.

Compared with other existing approaches, our method is more suitable for bearing degradation data with varying distributions and scales.

The remainder of the article is organized as follows. Section “Related works” gives the related work. In section “Proposed method” details the proposed DCSSL section “Experimental study” validates the proposed method on the FEMTO Bearing dataset. Section “Ablation studies” performs ablation studies. Section “Discussion and analysis” provides additional discussion and analysis of the experimental results. Finally, the conclusion is drawn in section “Conclusions”.

## Related works

### RUL prediction methods based on deep learning

Benefiting from the powerful nonlinear fitting capabilities, deep learning-based RUL prediction methods have gained significant attention in recent years. A variety of approaches have emerged, most of which leverage elaborately designed model architectures to learn degradation trends from large volumes of historical data, directly establishing a mapping from data to remaining useful time. Commonly adopted models include those based on CNN, LSTM, and GCN. For example, Zhang et al.^7^ proposed a CNN with training interference to process raw vibration signals, which replaces the manual representation extraction step, and the model performs well in noise environments. Lin et al.^8^ proposed an interpretable MP network which is trained in an end-to-end manner to achieve sparse time-frequency representations of vibration signals from gearboxes under variable speed conditions. Zhao et al.^9^ also employed an end-to-end approach, which aims to map directly from raw signals to RUL outputs via a neural network, utilizing a two-stage attention mechanism to extract input representations. Fu et al.^11^ designed an improved time series memory auto-encoder, which is capable of accurately reconstructing time series and reconstructing degradation trends. Shi et al.^12^ proposed an improved LSTM for predicting the RUL by characterizing the signal’s long-short time dependencies. Zhou et al.^15^ integrated the attention-based multi-perspective network with a temporal convolutional network to model temporal dependencies for RUL prediction. Qin et al.^16^ developed a Spatial-Temporal Multi-sensor Information Fusion Network for RUL, which effectively captures the spatial dependencies among multiple sensors. Qi et al.^17^ proposed a multi-task graph isomorphism network that incorporates a self-attention mechanism and a parameter-sharing mechanism to simultaneously perform fault diagnosis and remaining useful life prediction.

Wang et al.^18^ designed a gated graph convolutional network, combining GRU and GCN, to extract spatial-temporal graphs from multi-sensor data for RUL prediction. Among these, LSTM and GCN are more suitable for temporal data compared to CNN, as they can effectively capture temporal dependencies. However, they suffer from time-consuming training and high computational complexity.

In contrast to the aforementioned methods, our proposed DCSSL employed a novel architecture known as dilated causal convolution as the backbone network, which simultaneously possessed the capability to capture temporal dependencies while offering advantages such as low computational cost and fast training speed.

### Representation constraint strategies in RUL prediction

Early approaches primarily focused on architectural refinements of models, while overlooking the critical role of representation constraints. In RUL prediction, high-quality representations should exhibit characteristics such as smoothness and monotonicity^19^. To achieve this goal, researchers have conducted studies where a key strategy involves imposing constraints on the intermediate-layer representations during model learning. For example, Li et al.^20^ successfully extracted high-dimensional representations via a deep CNN using the sliding time window method. Zhu et al.^19^ proposed a contrastive learning method (CBHRL) using LSTM to capture continuous representations of bearing degradation data, which can be well mapped to RUL. Su et al.^21^ adopted a representation pre-extraction mechanism to replace the conventional representation fusion and representation selection operations, thereby further enhancing the model sensitivity to degraded representations. An et al.^22^ customized exclusive channels for representation vectors based on contrastive learning to make the extracted information more discriminative and to facilitate RUL prediction of bearings with incomplete lifecycle data. Liu et al.^23^ designed a model that can adaptively impose attention weights based on representation importance as a way to improve prediction performance.

In this study, the proposed DCSSL employed a novel SSL strategy that simultaneously performs contrastive learning at both the instance and timestamp levels. The instance-level contrast enables the model to capture object-specific characteristics, while the timestamp-level contrast allows it to learn temporal trends in degradation data, thereby extracting smooth representations highly correlated with the degradation process.

### SSL

SSL methods typically extract effective representations from large amounts of unlabeled data in pretext tasks, then transfer these representations to downstream tasks. It not only fully leverages unlabeled data to learn more robust representations but also offers the advantage of transferability^24–27^. For these reasons, researchers have become increasingly interested in SSL^28,29^ and have gradually begun to apply it to tasks such as classification, clustering, and prediction. For example, Kong et al.^30^ proposed an unlabeled sample learning (USL) architecture to optimize the performance of the model in RUL prediction through degradation information learned from unlabeled samples, and validation was accomplished on the dataset. He et al.^31^ utilized momentum contrastive learning to slowly update the encoder so that the learned representations are as consistent as possible. Chen et al.^32^ proposed a novel paradigm (SimCLR) for contrastive learning, demonstrating that the comparison task is helped to some extent by a variety of data augmentation operations and that the model performance is improved by adding a parameter borrowed from distillation temperature to the NT-Xent loss function. Chen et al.^33^ designed a progressive contrastive representation learning framework to address industrial surface defect detection in limited-label scenarios. Aaron van et al.^34^ proposed a generalized unsupervised method for extracting useful representations from high-dimensional data, employing noise contrast estimation as a loss function and using a similar approach to word embedding for end-to-end training. Wang et al.^35^ presented a self-supervised prediction method for the sample imbalance problem, which accomplishes fault detection through principal component analysis and error reconstruction strategies. Li et al.^36^ used a triplet loss to guide the model in updating the parameters, and then fine-tuned the offline model by letting the pseudo-labels update automatically, with performance close to that of supervised methods. Khosla et al.^37^ proposed a supervised contrastive method (SupCon), which leverages category labels to construct precise similarity constraints, extending contrastive learning to supervised tasks. Jathushan et al.^38^ first trained an optimal manifold using unlabeled data, and further constrained it by means of knowledge distillation to strengthen representational separability and improve the generalization capability of the few-shot classification model they designed.

Inspired by the SSL paradigm, we introduced dual-dimensional contrastive learning into the first learning stage of the proposed method to achieve representation learning. By participating in the RUL prediction training process in a representation-constrained manner, the interpretability of the model is enhanced, and its performance is significantly improved.

## Proposed method

### Problem definition

A set of *N* rolling bearing degradation data is given as ${\mathcal{D}_{{\mathrm{unlabeled}}}}=\{ {x_i}\} _{{i=1}}^{N}$, where each sample ${x_i} \in {{\mathbb{R}}^{T \times C}}$is a time series, *T* is the length of the time series (the number of timestamps), *C*is the representation dimension. Using a machine learning approach, a nonlinear representation function, i.e., representation extractor ${f_\theta }:{{\mathbb{R}}^{T \times C}} \to {{\mathbb{R}}^{T \times d}}$, is learned from ${\mathcal{D}_{{\mathrm{unlabeled}}}}$, and the loss function is minimized, which in turn yields a generalized representation extractor $f_{\theta }^{*}$, where *d* is the dimension of the representation for a single timestamp. The previously trained representation extractor$f_{\theta }^{*}$is then paired with a prediction head ${h_\phi }:{{\mathbb{R}}^{T \times d}} \to {{\mathbb{R}}^1}$ to make an RUL prediction for ${x_j}$ on another set of *M* rolling bearing degradation data ${\mathcal{D}_{{\mathrm{labeled}}}}=\{ {x_j},{y_j}\} _{{j=1}}^{M}$, and the prediction label ${\hat {y}_j}$is obtained. i.e. ${\hat {y}_j}={h_{{\phi ^*}}}\left( {{f_{\theta _{{{\mathrm{fin}}}}^{*}}}\left( {{x_j}} \right)} \right)$, where ${x_j} \in {{\mathbb{R}}^{T \times C}}$is identically distributed with ${x_i} \in {{\mathbb{R}}^{T \times C}}$ data, and finally compares the difference between ${\hat {y}_j}$and the true lifetime label ${y_j}$.

### DCSSL architecture

**Fig. 1:**
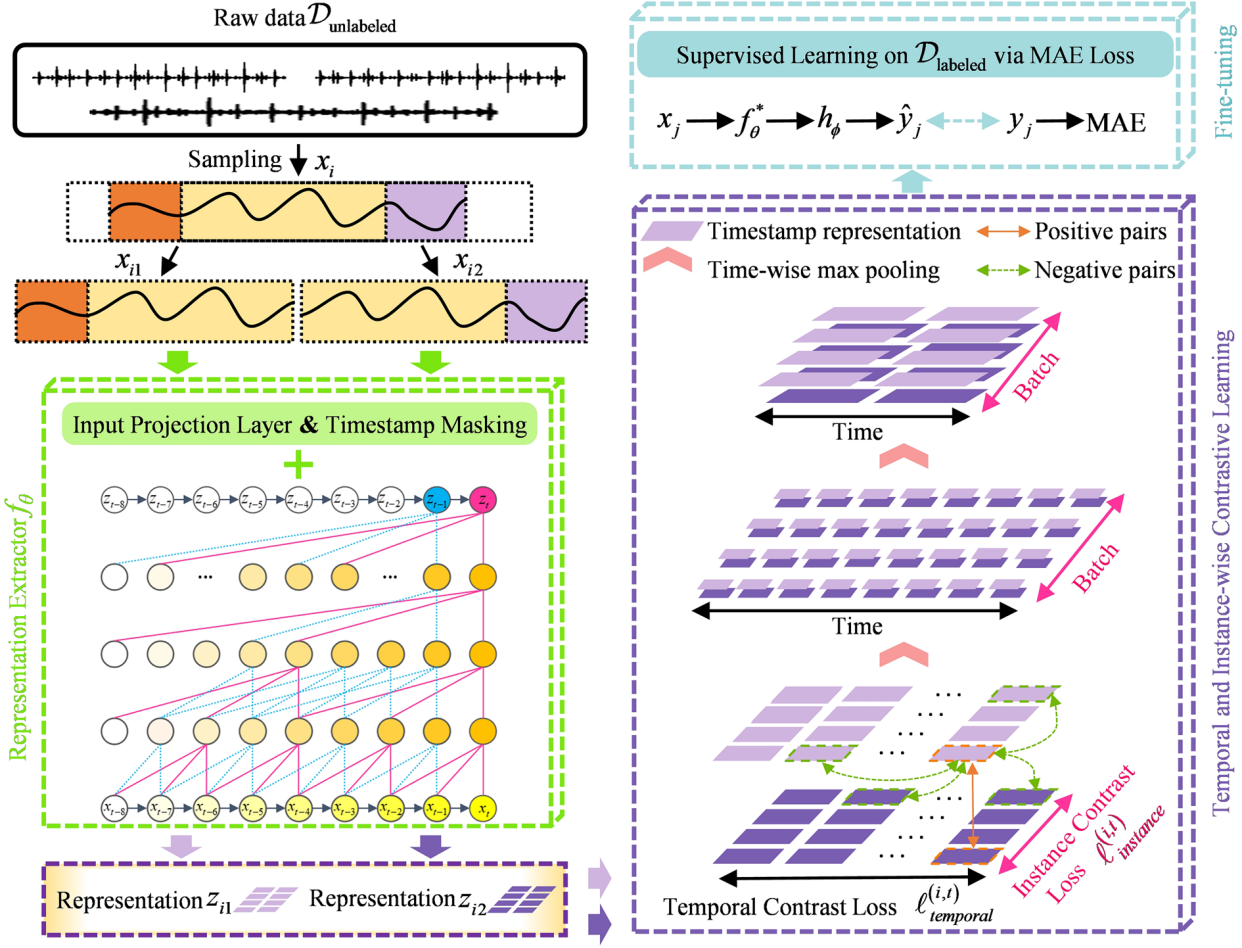
The framework of the DCSSL.

The overall DCSSL architecture is shown in Fig. 1. We first randomly extract two overlapping sub-segments ${x_{i1}}$ and ${x_{i2}}$ as augmented samples from ${x_i}$ on the rolling bearing degradation data ${\mathcal{D}_{{\mathrm{unlabeled}}}}=\{ {x_i}\} _{{i=1}}^{N}$, and input the raw data into the extractor ${f_\theta }$ used for representation extraction. In the next step, the degenerate value ${x_i}$ is mapped into the latent representation ${r_i}$ in the encoder through the input projection layer, using a mask operation to help mask the latent vector on the timestamp *t*. Subsequently, a dilated causal convolution is taken to fully extract the contextual representation by forcing the masked timestamp vector to be complemented by unmasked timestamp vectors, allowing the representation ${z_i}$ to be aligned in order to construct positive pairs for contrastive learning. This strategy enables the model to generate logically coherent and self-consistent representations based on the contextual information of the two augmented samples when processing the input data. Such interpretable representations allow the model to emphasize the ability to understand global information while also avoiding logical conflicts caused by narrow local understanding. It is worth mentioning that the extractor is made to combine temporal-level loss and instance-level loss for joint optimization. Finally, the trained $f_{\theta }^{*}$ is integrated into downstream tasks, where its extracted representations are used as input. The prediction head ${h_\phi }$ is fine-tuned using mean absolute error (MAE) to achieve precise RUL prediction.

### Dilated causal convolution

The dilated convolution can exponentially grow the size of the effective window without increasing the number of parameters or computational complexity, expand the coverage of the convolution kernel by using interval sampling, and then efficiently expand the context-aware range of the model^39^. As a strict temporal constraint model, causal convolution maintains temporal causality to ensure that when dealing with bearing vibration signals, the output of the model relies only on current and past information, not on future information, guaranteeing the temporal logic of faults and results^40^.

**Fig. 2:**
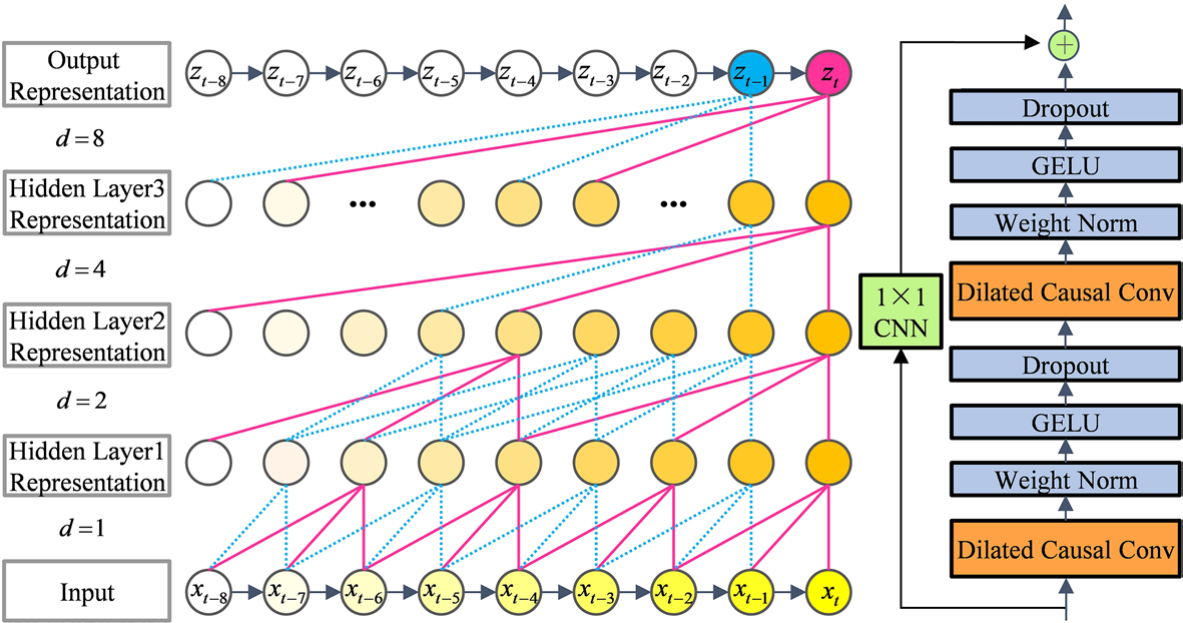
The architecture of the dilated causal convolution.

The dilated causal convolution combines the dilated sensory field of the dilated convolution with the temporal causality of the causal convolution. This combination allows the model to simultaneously capture long-term sequence dependencies and maintain strict temporal causality^41^. In this paper, a dilated causal convolution with eight residual blocks containing dilation parameters is used to extract a contextual representation of the vibration signal at each timestamp. From Fig. 2, it is clear that the output ${\hat {y}_t}$ of the model at timestamp *t* depends on the representations of the input ${x_t}$at timestampand the inputs before timestamp *t*. Similarly, the output${\hat {y}_{t - 1}}$at timestamp$t - 1$depends on the representations of the input at timestamp$t - 1$and at all previous timestamps. It uses the pointwise convolution in each residual network to ensure that input-output dimensions of the model remain consistent. And the expansion parameter *d* ($d={2^i},i=\left\{ {1,2,3, \cdot \cdot \cdot ,8} \right\}$) corresponding grows sequentially.

### The strategy for constructing positive pairs

**Fig. 3:**
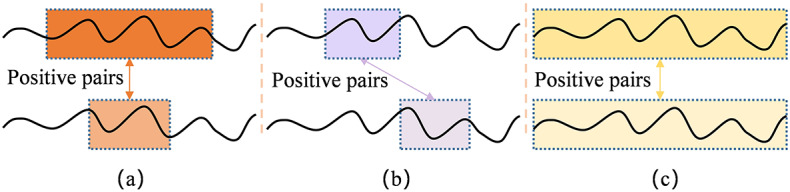
Different strategies for constructing positive pairs. (a) Subsequence consistency. (b) Temporal consistency. (c) Transformation consistency.

Figure 3 illustrates several positive pairs construction strategies designed for temporal data such as bearing degradation data, where (a) denotes subsequence consistency (orange color), (b) represents temporal consistency (purple color), and (c) corresponds to transformation consistency (yellow color). Although these methods can improve the model performance, there are limitations, such as localized phase shifts, delays in the response to sudden failures, and effective signal distortion.

**Fig. 4:**
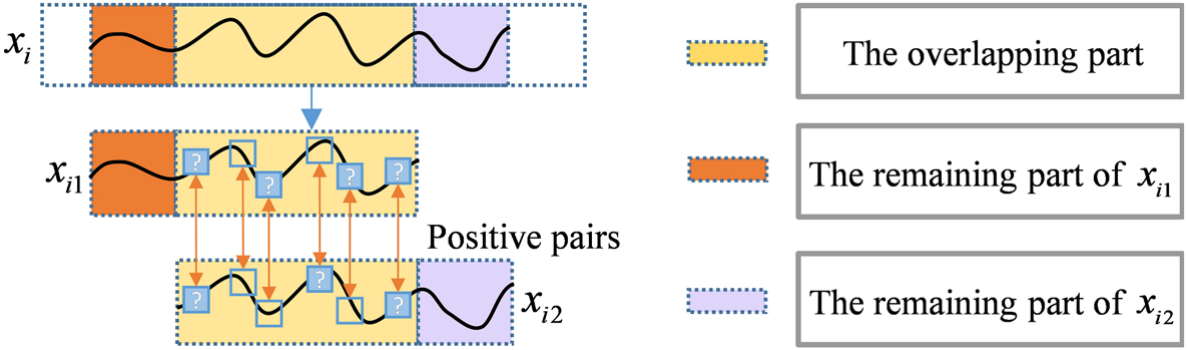
The proposed strategy for constructing positive pairs.

The proposed strategy in Fig. 4 improves the robustness of the representations through random cropping and timestamp masking of the context, which highlights the logical self-consistency. Concretely speaking, for any segment of vibration data ${x_i} \in {{\mathbb{R}}^{T \times C}}$, two overlapping segments${x_{i1}}$and${x_{i2}}$are randomly sampled by cropping, and the overlapping signal segments in the two augmented samples have consistent representations.

For a particular segment of augmented vibration samples, we further replace the randomly selected timestamps with mask identifiers to enhance the understanding of local contexts. In other words, we aim to force the model to understand the smooth transition law of vibration signals at adjacent time points, relying on the context to infer the masked part. So that positive pairs with causal associations can be constructed. With the assistance of this strategy, the model is able to strengthen the ability to correlate local segments with the global degradation stage and enhance the robustness of predictions under localized failure modes.

### The dual-dimensional contrast

To better apply contrastive learning to the tasks of bearing RUL prediction, based on the construction of the strategy, we propose a dual-dimensional contrastive loss function, where the temporal-level contrast is used to model the continuous evolution of the degradation state within the same bearing, while the instance-level contrast aims to mitigate the differences in degradation patterns between different bearing individuals.

To learn the discriminative representation of the same vibration signal over time, a segment of the vibration signal ${x_i}$ is randomly cropped to obtain a pair of augmented samples (${x_{i1}}$, ${x_{i2}}$). Taking the representation ${z_{i1,t}}$ of ${x_{i1}}$ at the timestamp *t* as an example, in this paper, we consider the representation ${z_{i2,t}}$ of ${x_{i2}}$ at the same timestamp as a positive sample, and consider the representations ${z_{i1,n}}$ and ${z_{i2,n}}$ corresponding to ${x_{i1}}$ and ${x_{i2}}$ at different timestamps (e.g., at *n*) respectively as negative samples. The model performs max pooling along the time axis on the learned representations. The contrastive loss function at the temporal-level is as follows:

1 $$\ell _{{temporal}}^{{\left( {i,t} \right)}}= - \log \frac{{\exp \left( {{z_{i1,t}} \cdot {z_{i2,t}}} \right)}}{{\sum {_{{_{{n \in \Omega }}}}\left( {\exp \left( {{z_{i1,t}} \cdot {z_{i2,n}}} \right)+{{\mathbb{I}}_{\left[ {t \ne n} \right]}}\exp \left( {{z_{i1,t}} \cdot {z_{i1,n}}} \right)} \right)} }}$$

where $\Omega$ is the set of timestamps of the overlapping parts of the local segments of the two augmented vibration signals, ${\mathbb{I}}$ represents the indicator function, which takes the value 1 when $t \ne n$ and 0 otherwise.

Similarly, to learn the differential representation of different vibration signals under the same batch *B*, two segments of vibration signals ${x_i}$ and ${x_j}$ are randomly cropped, respectively, and the representation ${z_{i2,t}}$ is regarded as a positive sample for the representation ${z_{i1,t}}$, and the representations ${z_{j1,t}}$ and ${z_{j2,t}}$ corresponding to ${x_{j1}}$ and ${x_{j2}}$ are regarded as negative samples. The contrastive loss function at the instance-level is as follows:

2 $$\ell _{{instance}}^{{\left( {i,t} \right)}}= - \log \frac{{\exp \left( {{z_{i1,t}} \cdot {z_{i2,t}}} \right)}}{{\sum {_{{_{{j=1}}}}^{B}\left( {\exp \left( {{z_{i1,t}} \cdot {z_{j2,t}}} \right)+{{\mathbb{I}}_{\left[ {i \ne j} \right]}}\exp \left( {{z_{i1,t}} \cdot {z_{j1,t}}} \right)} \right)} }}$$

The dual-dimensional contrast is indispensable to capture both the relevant representations of the bearing vibration signals and to tap into the dynamic trend of bearing degradation. Further introducing the weighting hyper-parameter $\beta$, the total loss is defined as:

3 $${\mathcal{L}_{dual}}=\frac{1}{{NT}}\sum\limits_{i} {\sum\limits_{t} {\left( {\beta \ell _{{temporal}}^{{\left( {i,t} \right)}}+\left( {1 - \beta } \right)\ell _{{instance}}^{{\left( {i,t} \right)}}} \right)} }$$

Finally, to ensure logical coherence throughout the representation learning stage of the framework, the complete pseudo-code is presented in Algorithm 1.

### Model fine-tuning

Through the previous learning stages, a trained representation extractor $f_{\theta }^{*}$ has been obtained. To enable the model to ultimately predict RUL, a new prediction head ${h_\phi }$ is created and fine-tuned using mean absolute error (MAE). For each input sample, the prediction head uses the representations extracted by $f_{\theta }^{*}$ as input and outputs the RUL.

**Figure:**
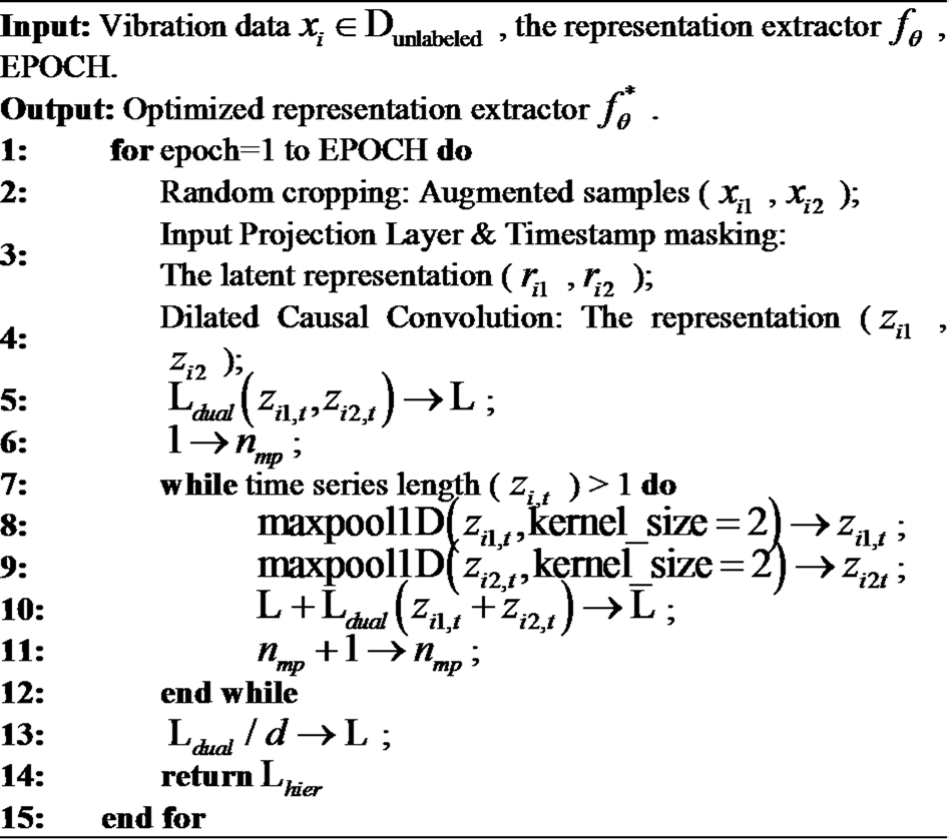
**Algorithm 1** Representation learning algorithm for DCSSL.

## Experimental study

### Data description

To validate the performance of the DCSSL, research and analysis were conducted on the FEMTO Bearing dataset to demonstrate its effectiveness and superior performance.

**Fig. 5:**
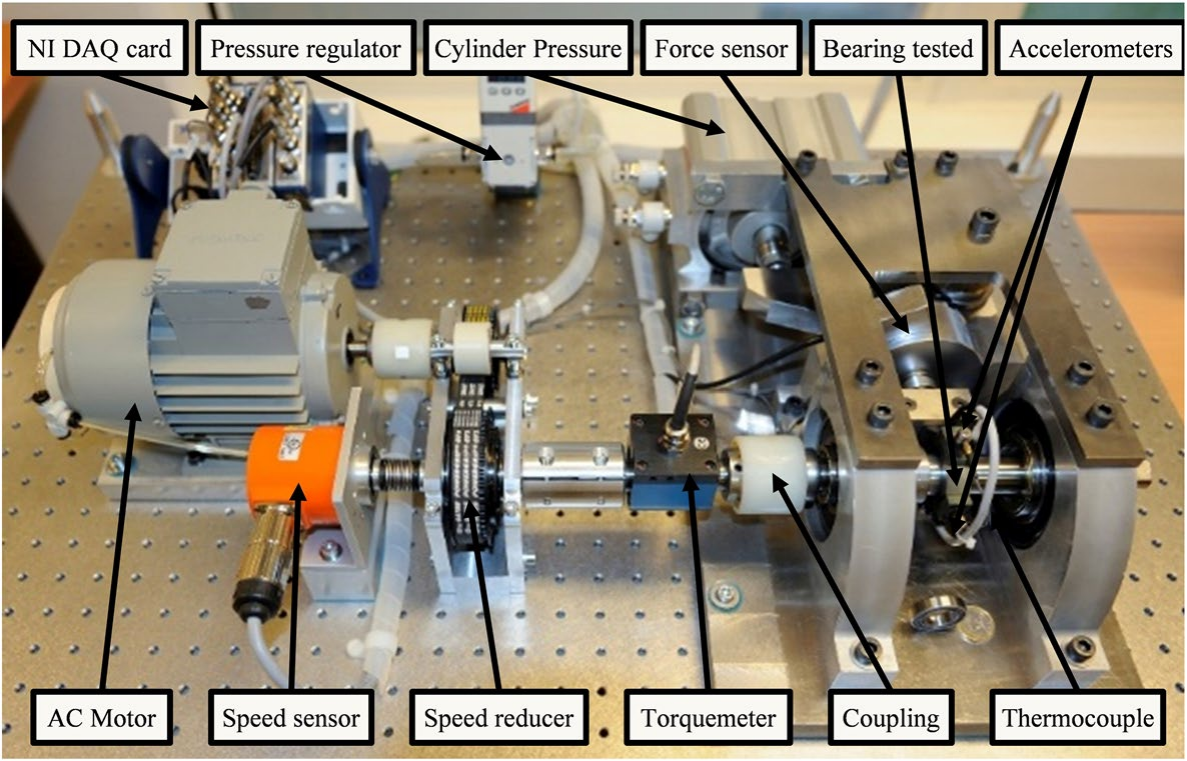
Experimental platform for the FEMTO Bearing dataset^42^.

This work utilizes the dataset from the IEEE PHM 2012 Data Challenge, which was jointly organized by the IEEE Reliability Society and the FEMTO-ST Institute^42^. (https://phm-datasets.s3.amazonaws.com/NASA/10.+FEMTO+Bearing.zip). It was collected using a dedicated accelerated degradation test platform PRONOSTIA, as shown in Fig. 5, which simulates the operating conditions of bearings under variable speed and load. Its main objective is to provide real experimental data to describe the degradation of roller bearings during their complete lifecycles, which consists of three main components: the rotating part, the loading part, and the measuring part.

The data content includes acceleration sensor data in the horizontal and vertical directions at a 25.6 kHz sampling rate employing cyclic acquisition of 0.1 s recordings per 10 s, and 2560 samples recorded. There are 17 sets of complete test data, of which 6 sets of data were provided with RUL real labels and run complete to failure, i.e., full lifecycle, and used for training, and 11 sets used for testing were truncated halfway without labels. Each data set contains millions of sampling points, stored in units of 10 s per segment.

### Experiment setup

During the experiment preparation phase, we use the sliding window method to generate training and testing samples. Specifically, we divide the vibration data (the whole time sequence) of a bearing into multiple time windows, i.e., we use the window with a fixed size to sweep through the whole bearing vibration signal, and crop the corresponding overlapped data points, which is treated as input sample and then assigned with a RUL label. Table 1 specifies the sources of the training and testing data. The dataset provides run-to-failure data from six bearings under three operating conditions to construct the prediction model, while also providing data from the remaining 11 bearings for RUL prediction. Taking Condition 1 as an example, Bearing1_1 and Bearing1_2 are used for model training, and Bearing1_3 through Bearing1_7 are used for testing. To brief the experimental protocol, in the representation phase of DCSSL, the unlabeled dataset ${\mathcal{D}_{{\mathrm{unlabeled}}}}$ comprises the inputs of all training samples; subsequently, during the fine-tuning phase, the labeled dataset ${\mathcal{D}_{{\mathrm{labeled}}}}$incorporates the inputs along with their corresponding labels of all training samples.

**Table 1:** Division of the dataset.

Dataset	Condition 1 1800 (rpm), 4000 (*N*)	Condition 2 1650 (rpm), 4200 (*N*)	Condition 3 1500 (rpm), 5000 (*N*)
Training bearings	1_1 & 1_2	2_1 & 2_2	3_1 & 3_2
Testing bearings	1_3	2_3	3_3
	1_4	2_4	
	1_5	2_5	
	1_6	2_6	
	1_7	2_7	

In the field of RUL prediction of mechanical systems, the selection of evaluation indices directly affects the objectivity of model performance. In this research, aimed at investigating the prediction accuracy of DCSSL, mean square error (MSE) and MAE were used to evaluate the effect, and their definitions and formulas are as follows:

4 $$\begin{gathered} {\mathrm{MSE}}=\frac{1}{n}\sum\limits_{{i=1}}^{n} {{{\left( {{y_i} - {{\hat {y}}_i}} \right)}^2}} \hfill \\ {\mathrm{MAE}}=\frac{1}{n}\sum\limits_{{i=1}}^{n} {\left| {{y_i} - {{\hat {y}}_i}} \right|} \hfill \\ \end{gathered}$$

Experimental computations were performed on a PC equipped with an AMD Ryzen 9 5900 CPU, 32GB of DDR4 RAM, and an NVIDIA GeForce RTX 3080 GPU. The hyper- parameters are displayed in Table 2.

**Table 2:** List of model hyper-parameters for DCSSL.

Hyper-parameter	Value
Timesteps	20
Batchsize	256
Output_dims	1024
Hidden_dims	32
Depth	8
$\tau$	0.07
$\beta$	0.3

### Experimental results and comparisons with other relevant deep learning methods

To demonstrate the superiority of DCSSL, by comparing its performance with four mainstream RUL prediction methods, namely InfoTS^43^, USL^30^, CBHRL^19^, SimCLR^32^, and SupCon^37^, on the FEMTO Bearing dataset, as detailed in Table 3.

As evidenced by Table 3, DCSSL achieves superior MSE performance over other state-of-the-art methods in 6 groups out of 11 bearing RUL prediction experiments. For Bearing1_7, DCSSL yields an MSE of 0.0009 compared to 0.0015 for InfoTS, 0.0011 for USL, 0.0052 for CBHRL, 0.0021 for SimCLR, and 0.0038 for SupCon. DCSSL attains second-best performance in Bearing2_3. Overall, in terms of average performance, DCSSL is still 7.64% better than the second-best method, InfoTS. These results reflect that the DCSSL method is more capable of controlling the extreme prediction errors, such as early misclassification or near-failure misreporting, and it can significantly improve the prediction stability.

**Fig. 6:**
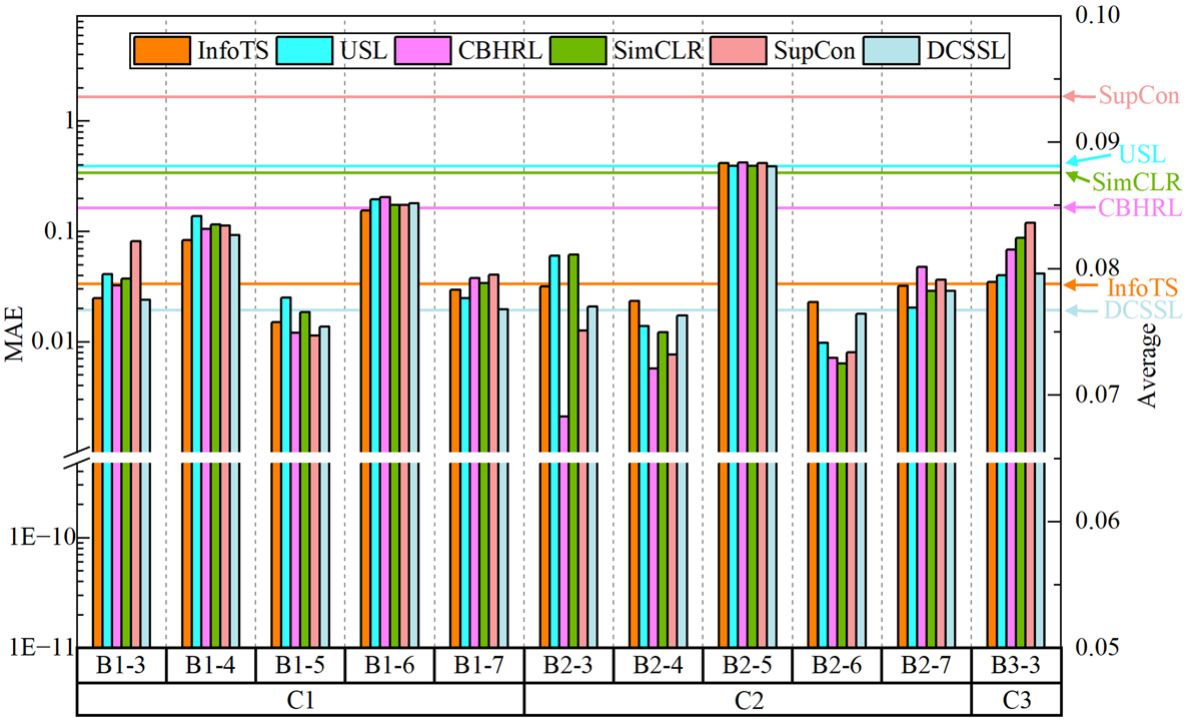
Comparison of the MAE of different methods on each bearing sample.

As can be seen from Fig. 6, the results of the compared experiments of bearing RUL prediction are presented. DCSSL achieves lower MAE values than other advanced methods in 5 groups out of 11 experiments, as compared to other methods, which achieve the best performance in only 3 groups at most. Taking Bearing1_7 as an example, the MAE value of DCSSL is 0.0195, while the MAE values of InfoTS, USL, CBHRL, SimCLR, and SupCon are 0.0294, 0.0247, 0.0377, 0.0338, and 0.0404, respectively. The average MAE performance of each method is highlighted in the figure. It can be seen that DCSSL is 2.17% ahead of the second-best InfoTS. The lower MAE value proves that the RUL prediction result is closer to the true failure time and that the distribution of deviations is highly centralized and more robust, reflecting the improvement of DCSSL in overall predictive reliability, which can help develop accurate maintenance strategies.

**Table 3:** Comparison with other advanced methods under MSE.

Training bearings	Testing bearings	Method					
		InfoTS	USL	CBHRL	SimCLR	SupCon	DCSSL
1_1 & 1_2	1_3	0.0037	0.0031	0.0040	0.0030	0.0213	**0.0011**
	1_4	0.0566	0.0805	0.0569	0.0560	0.0576	**0.0476**
	1_5	0.0047	0.0014	0.0049	0.0006	0.0046	**0.0005**
	1_6	0.1003	0.1095	0.0820	0.0904	**0.0735**	0.0892
	1_7	0.0015	0.0011	0.0052	0.0021	0.0038	**0.0009**
2_1 & 2_2	2_3	0.0052	0.0449	**0.0005**	0.1849	0.0150	0.0027
	2_4	**0.0012**	0.0029	0.0016	0.0024	0.0017	0.0014
	2_5	0.2577	0.2565	0.2782	0.2577	0.2752	**0.2538**
	2_6	**0.0010**	0.0028	0.0018	0.0013	0.0014	0.0012
	2_7	0.0107	0.0080	0.0229	0.0089	0.0117	**0.0075**
3_1 & 3_2	3_3	**0.0044**	0.0097	0.0091	0.0341	0.0619	0.0068
Avg.		0.0406	0.0473	0.0425	0.0583	0.0480	**0.0375**

**Fig. 7:**
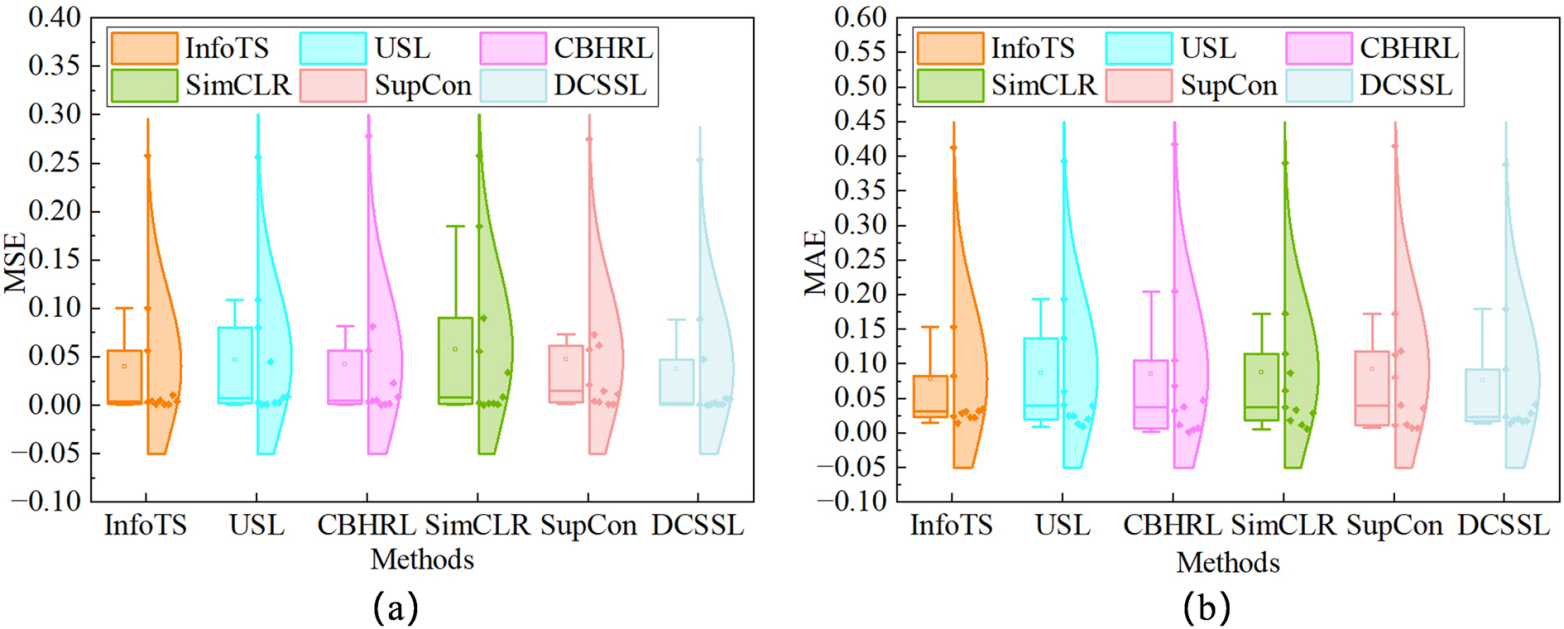
Distribution of MSE and MAE for different methods.(a) Distribution of MSE for InfoTS, USL, CBHRL, SimCLR, SupCon and DCSSL.(b) Distribution of MAE for InfoTS, USL, CBHRL, SimCLR, SupCon and DCSSL.

The analysis of MAE and MSE boxplots in Fig. 7 shows that DCSSL exhibits significant advantages in prediction accuracy and robustness. First, its median MAE and MSE are lower than all other compared methods, proving that the prediction bias is smaller on 50% of the test samples. Second, the interquartile range (IQR) of DCSSL is the narrowest among all the methods, which indicates that it is less sensitive to the condition perturbation and noise and has stronger prediction stability. These indicate that DCSSL not only reduces the average prediction error but also increases the reliability of the bearing RUL prediction results.

**Fig. 8:**
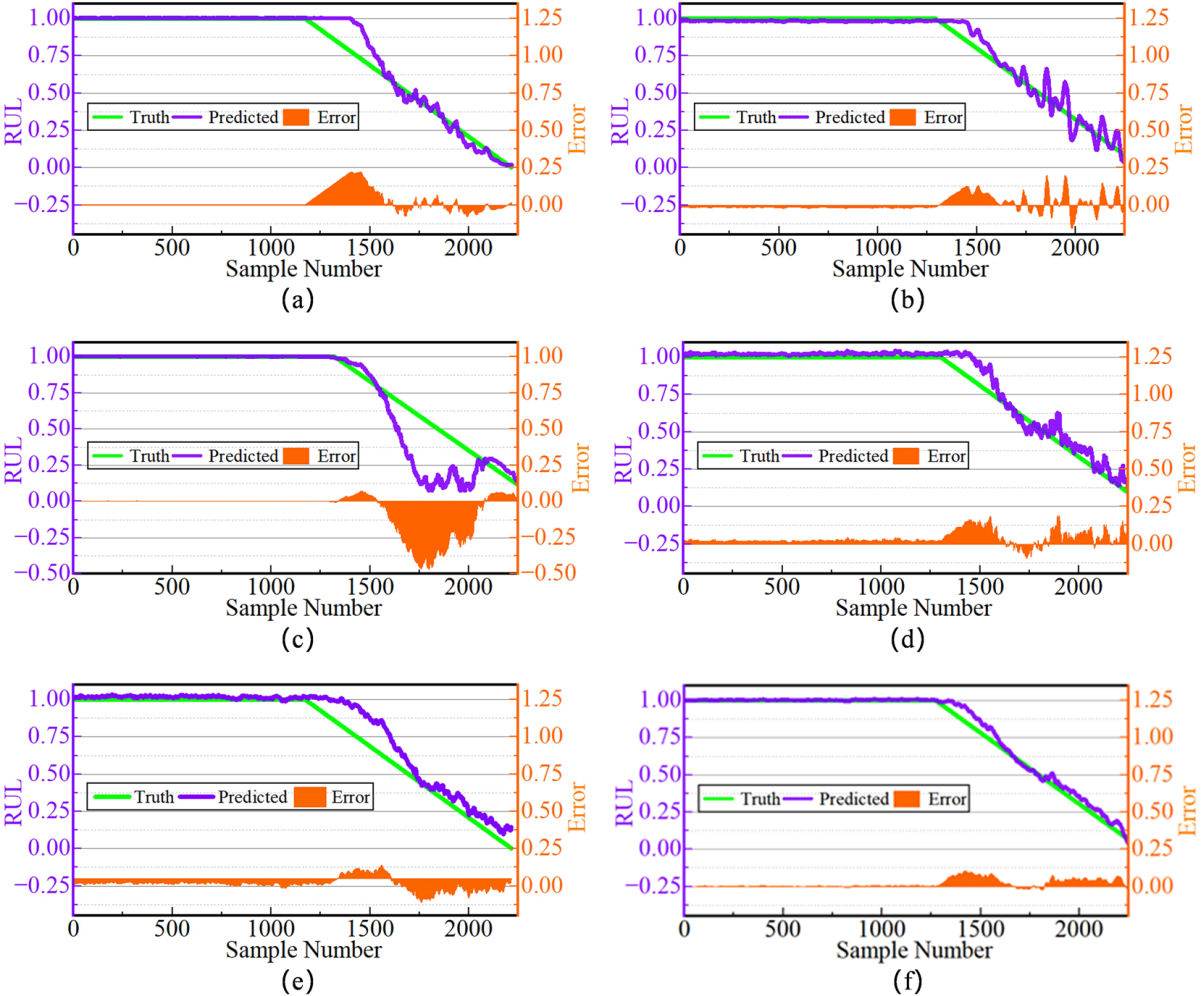
Prediction effect of compared methods on Bearing1_3. (a) Prediction effect of CBHRL on Bearing1_3. (b) Prediction effect of SimCLR on Bearing1_3. (c) Prediction effect of SupCon on Bearing1_3. (d) Prediction effect of USL on Bearing1_3. (e) Prediction effect of InfoTS on Bearing1_3. (f) Prediction effect of DCSSL on Bearing1_3.

**Fig. 9:**
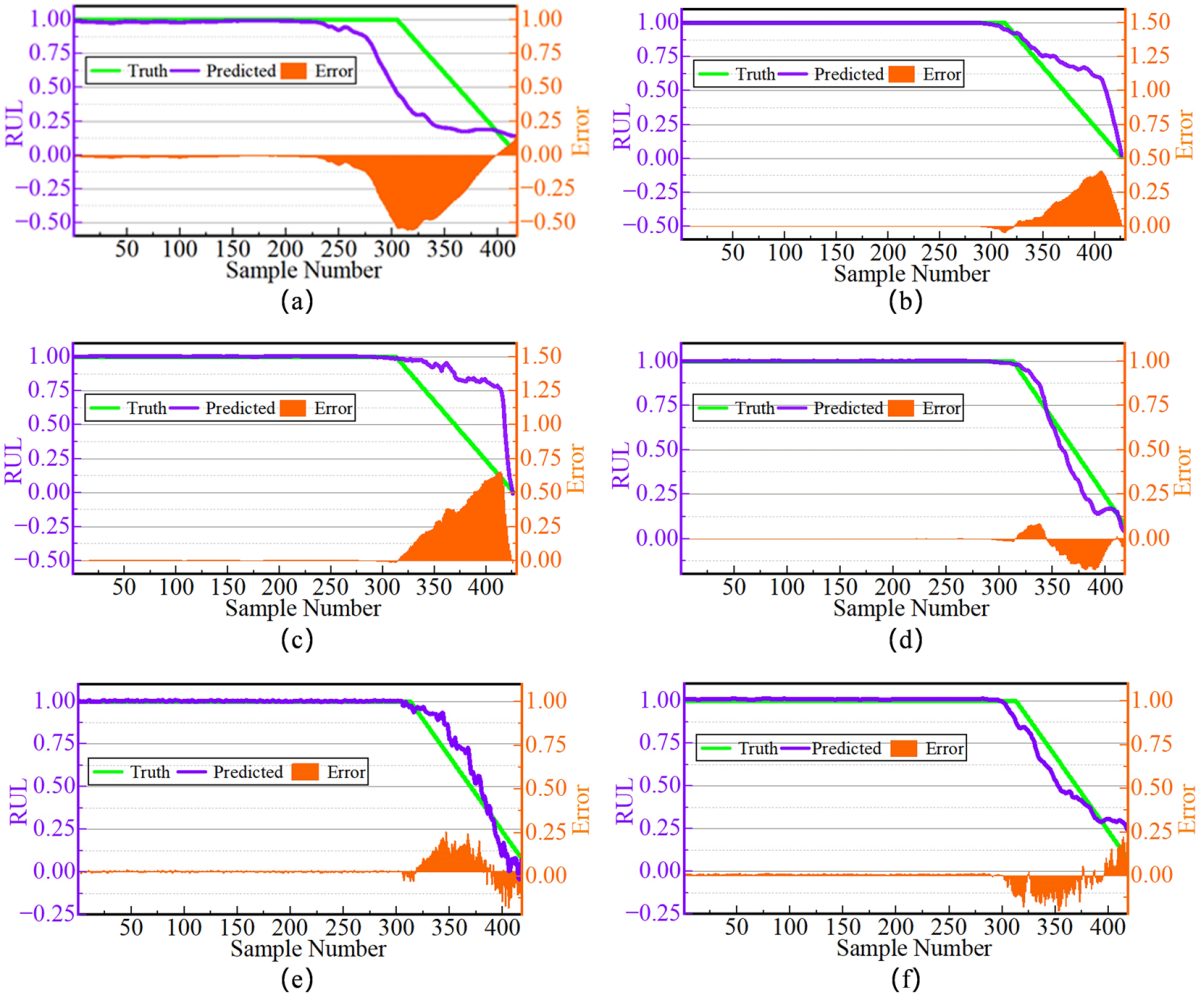
Prediction effect of compared methods on Bearing3_3. (a) Prediction effect of CBHRL on Bearing3_3. (b) Prediction effect of SimCLR on Bearing3_3. (c) Prediction effect of SupCon on Bearing3_3. (d) Prediction effect of USL on Bearing3_3. (e) Prediction effect of InfoTS on Bearing3_3. (f) Prediction effect of DCSSL on Bearing3_3.

The RUL curve of bearings fundamentally represents the process of cumulative material damage and progressive performance degradation. Taking the RUL prediction curves of bearings 1_3 and 3_3 in Figs. 8 and 9 as examples, the rate of bearing degradation during the healthy stabilization period is slow, the vibration fluctuations are weak, and the slopes of the curves tend to be close to zero. Subsequently, the accelerated degradation phase manifests an exponential decay in RUL until functional failure. Ultimately, at the failure-critical stage, vibration amplitude exhibits significant excursions proximate to the failure threshold. Furthermore, whether in the initial stage of slow change, the middle stage of accelerated degradation, or the final stage of sudden failure, the decay trends predicted by DCSSL are more consistent with actual observations, in sharp contrast to situations where degradation is triggered too early or too late.

In comparison, other methods show significant deviations in deterioration rate estimation, which can be attributed to the innovations of DCSSL in degradation representation extraction, positive pairs construction strategy, and loss function design. In addition, it can also be seen that the error curves of DCSSL are smoother, less fluctuating, and free of sudden changes, and these results fully demonstrate the excellent effectiveness of DCSSL.

## Ablation studies

### Impact of model structural components on the model

During the design process of the proposed model, we incorporated three essential components: random cropping to address spatial variability, timestamp masking for temporal continuity, and an input projection layer enhancing the representation discriminability of bearing degradation signals. Ablation studies confirm their necessity in Table 4: Removing random cropping causes overfitting to position-specific fault patterns during training, impairing generalization to identical faults emerging at new locations and significantly increasing RUL prediction errors. Eliminating timestamp masking degrades the model capability to infer degradation onset from sparse anomalies, resulting in delayed early-stage RUL predictions. Discarding the input projection layer prevents effective separation of noise from degradation representations, distorting degradation trajectory representation, and severely reducing prediction accuracy for RUL inflection points and degradation rates. Therefore, all three components provide unique and synergistic contributions in addressing rolling bearing signal representations and enhancing prediction robustness.

**Table 4:** Impact of model structural components on the model.

Component	MSE	RMSE	MAE	${{\mathrm{R}}^2}$
Random cropping	0.0103	0.1017	0.0502	0.8538
Timestamp masking	0.0423	0.2056	0.1005	0.4017
Input projection layer	0.0109	0.1045	0.0498	0.8454
**DCSSL**	**0.0067**	**0.0822**	**0.0408**	**0.9045**

### Impact of loss function combinations on the model

To evaluate the impact of different loss functions on model performance, ablation studies in Table 5 shows that: The instance-level contrastive loss focuses on learning the differences in degradation states between different bearing samples, which can effectively identify the health and fault states of the bearings, but is unable to model the continuous and gradual representations of the degradation trajectories, resulting in insufficient early fault sensitivity, and even performance is not as good as that of the model that does not use the contrastive loss. The temporal-level contrastive loss imposes temporal continuity constraints within individual bearing samples. While this enables the model to capture gradual progression patterns of fault representations, its limited cross-sample discriminative capability causes confusion between early-stage faults and noise-corrupted samples. The model lacking the dual-dimensional contrastive loss struggle to extract consistent degradation representations from vibration signals due to insufficient self-supervised representation learning. The model using the dual-dimensional contrastive loss approach delivered superior results. It can ensure separability of degradation states and smoothness of evolutionary trajectories by synergistically optimizing the two losses, providing discriminative and continuous representations for RUL prediction.

**Table 5:** Impact of loss function combinations on the model.

Loss function	MSE	RMSE	MAE	${{\mathrm{R}}^2}$
W/o temporal contrast	0.0163	0.1276	0.0583	0.7697
W/o instance contrast	0.0075	0.0948	**0.0401**	0.8942
W/o contrastive loss	0.0090	0.0948	0.0488	0.8728
**DCSSL**	**0.0067**	**0.0822**	0.0408	**0.9045**

### Impact of positive pairs construction strategy on the model

To validate the superiority of our proposed strategy for constructing positive pairs, comparative experiments were conducted against subseries consistency and temporal consistency approaches. The results in Table 6 demonstrate that model using the contextual consistency strategy achieve the best performance by accurately capturing the physical principles of bearing degradation, seamlessly combining local anomaly detection and global evolution modeling within vibration signals. Subseries consistency destroys the temporal causality of the vibration signals by forcing the alignment of random segments, leading to dilution of the incipient fault indicator during random cropping. Temporal consistency imposes excessive similarity constraints on adjacent time points, confusing representations between abrupt operational changes and genuine degradation. Thus, both methods underperform.

**Table 6:** Impact of positive pairs construction strategy on the model.

Strategy	MSE	RMSE	MAE	${{\mathrm{R}}^2}$
Subseries consistency	0.0069	0.0833	**0.0384**	0.9018
Temporal consistency	0.0081	0.0901	0.0430	0.8852
**DCSSL**	**0.0067**	**0.0822**	0.0408	**0.9045**

### Impact of additional data augmentation approaches on the model

In this section, we investigate the impact of other data augmentation approaches on the model to construct positive and negative pairs. By introducing jitter, scaling and permutation into the base model to construct the enhanced positive pairs, the experiment results in Table 7 show that the early degradation representations of bearings are weak in the original data, while jitter or scaling can simulate the real disturbances, but may also drown out these key representations, which will weaken the ability of the model to capture the faults: The early degradation representations of bearings are relatively weak in the original data, and although jitter or scaling can simulate the real disturbances, they may also drown out these key representations, which in turn weakens the ability of the model to capture the faults; permutation has the possibility of destroying the temporal structure of the bearing data, which will lead to the model learning the wrong evolution law at the temporal-level. The bearing inspection data usually has a high signal-to-noise ratio and consistency, and the direct use of raw or simply cropped signals can maximize the retention of the subtle representations of early degradation and the exponential growth trend of fault representation amplitude. The dual-dimensional contrastive learning proposed in this paper already provides sufficient sample diversity, and additional enhancement methods would instead lead to a decrease on model performance^44^.

**Table 7:** Impact of additional data augmentation approaches on the model.

Augmentation	MSE	RMSE	MAE	${{\mathrm{R}}^2}$
Jitter	0.0147	0.1212	0.0566	0.7923
Scaling	0.0158	0.1256	0.0578	0.7769
Permutation	0.0553	0.2351	0.1116	0.2183
**DCSSL**	**0.0067**	**0.0822**	**0.0408**	**0.9045**

### Impact of the backbone network on the model

To validate the rationality of backbone network selection, ablation studies replaced the dilated CNN with LSTM and Transformer under comparable parameter constraints. Results in Table 8 demonstrate that dilated CNN achieves linear receptive field expansion: through convolutional kernels and dilation mechanisms, efficiently capturing local degradation representations in bearings. Its inherent architectural properties substantially mitigate overfitting risks while enhancing generalization capability, making it ideally suited for bearing RUL prediction tasks. LSTM’s temporal recurrence dilutes localized representations of millisecond-scaled fault impacts in bearing vibration signals. Its sequential sensitivity disrupts the time-shift invariance essential for fault identification, conflicting with bearing degradation physics. Transformer’s self-attention, due to excessive focus on global dependencies, amplifies noise while diluting transient fault representations, limiting generalization of learned degradation patterns.

**Table 8:** Impact of the backbone network on the model.

Backbone	MSE	RMSE	MAE	${{\mathrm{R}}^2}$
LSTM	0.0107	0.1033	0.0553	0.8491
Transformer	0.0069	0.0833	0.0439	0.9017
**DCSSL**	**0.0067**	**0.0822**	**0.0408**	**0.9045**

## Discussion and analysis

### Time window size

**Fig. 10:**
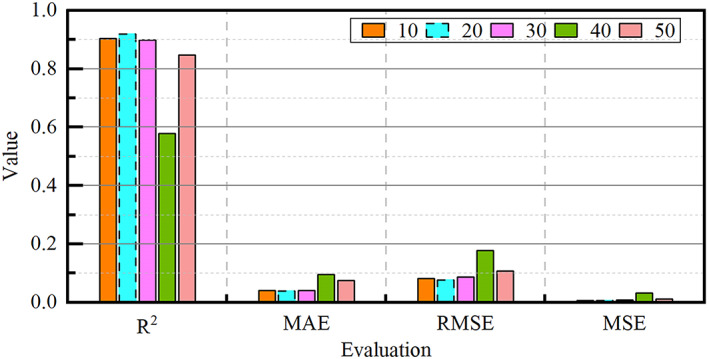
Evaluation indicators under different time window sizes.

During the design Results in Fig. 10 demonstrate the impact of different time window sizes on the model performance. When the time window size increases from 10 to 20, the model performance reaches the optimum, which can accurately capture the key representations of bearing degradation, at which time${{\mathrm{R}}^2}=0.9194$. Subsequently, the model performance decreases with the increase in the window size.

The choice of window size essentially corresponds to the time scale of bearing degradation; too small a size cannot cover the complete fault development cycle, resulting in the loss of early weak fault representations, and certain noise disturbances may be misclassified as degradation signals, decreasing the reliability of positive pairs established by comparative learning. With too large a size, the window may contain data from different stages, and this redundant historical information will weaken the model sensitivity to the onset of degradation, leading to distorted prediction results.

### Number of model layers

Results in Table 9 demonstrate the impact of the number of model layers on the model performance, where the model performance reaches its optimum when the number of model layers is increased from 2 to 8, at which time${{\mathrm{R}}^2}=0.9194$. Subsequently, when the number of model layers is increased to 10, the performance decreases instead. When the number of model layers is shallow, the model fails to converge on the loss function due to insufficient representation capability, and fails to establish a nonlinear trajectory from normal to wear and finally to failure. When the number of model layers is deeper, the gradient back propagation path is too long, and the parameters near the input layer stop updating, which affects the ability of the model to learn early degradation representations, and also ignores the degradation law that should have been learned because of too much focus on the noise, which ultimately leads to overfitting.

**Table 9:** Impact of the number of model layers on the model.

Depth	MSE	RMSE	MAE	${{\mathrm{R}}^2}$
2	0.0115	0.1074	0.0480	0.8396
4	0.0096	0.0978	0.0468	0.8670
6	0.0093	0.0967	0.0474	0.8701
**8**	**0.0058**	**0.0762**	**0.0396**	**0.9194**
10	0.0072	0.0850	0.0460	0.8996

### Output representation dimension

The output representation dimension refers to the length of the representation extracted by the dilated causal convolution for each timestamp. We further investigated the impact of this dimension on final RUL prediction under different values. Results in Table 10 demonstrate the impact of output representation dimension on the model performance, with the model reaching optimal performance when the dimension is increased from 16 to 1024.

Higher dimension can provide a more comprehensive description on the state of a timestamp, enabling the model to more accurately capture bearing health conditions. This facilitates the successful learning of continuous, smooth representations of bearing degradation, ultimately achieving more precise RUL predictions. Furthermore, high dimension space transforms the originally nonlinear problem into one that is approximately linearly separable. The resulting linear decision boundary ensures both the interpretability and generalization capability of the model.

**Table 10:** Impact of output representation dimension on the model.

Dimension	MSE	RMSE	MAE	${{\mathrm{R}}^2}$
16	0.0339	0.1842	0.0958	0.5284
32	0.0107	0.1032	0.0479	0.8520
64	0.0126	0.1124	0.0513	0.8245
128	0.0140	0.1181	0.0553	0.8061
256	0.0058	0.0762	**0.0396**	0.9194
512	0.0054	0.0737	0.0419	0.9246
**1024**	**0.0054**	**0.0735**	**0.0435**	**0.9248**

### Hidden layer dimension

The hidden layer dimension refers to the channel size of each dilated causal convolution. We further investigated the impact of this dimension on final RUL prediction under different values. Results in Table 11 show the impact of hidden layer dimension on the model performance. When the number of layers of the model is 32, the model performance reaches its optimum, at which time${{\mathrm{R}}^2}=0.9376$. It demonstrates that the current model complexity is optimal for the experimental task. As the number of layers continues to increase, the model performance begins to fluctuate and drops sharply beyond 256 layers. This indicates that excessive hidden layer dimension may cause the model to overly focus on noise rather than capture the underlying degradation pattern. Furthermore, such a deep network also introduces gradient instability during backpropagation.

**Table 11:** Impact of hidden layer dimension on the model.

Dimension	MSE	RMSE	MAE	${{\mathrm{R}}^2}$
**32**	**0.0045**	**0.0670**	**0.0385**	**0.9376**
64	0.0062	0.0786	0.0448	0.9142
128	0.0054	0.0735	0.0435	0.9248
256	0.0563	0.2372	0.1153	0.2182
512	0.0704	0.2653	0.1932	0.0219

### 6.5 Hyper-parameter$\boldsymbol{\boldsymbol{\upbeta}}$

To explore the impact of the hyper-parameter $\beta$in the dual-dimensional contrastive loss function on the model performance, $\beta$ is gradually increased from 0.1 to 0.9 to test the results, and the results are shown in Table 12.

As can be seen from the table, the best prediction result is achieved when $\beta$ is 0.3, and ${{\mathrm{R}}^{\mathrm{2}}}$at this time is 0.9497. In terms of weight distribution, this is the same as the temporal distribution of the bearing degradation process, i.e., about 70% of the time in the whole life cycle is the smooth degradation period, and about 30% of the time is the healthy-to-failure leap period. From the perspective of loss function optimization, the loss gradient at the temporal-level dominates the local smoothness of the bearing degradation trajectory, and the loss at the instance-level guarantees the distinguishability of the bearing health state; and the two are in dynamic equilibrium at this point. From the perspective of decision preference, the dual-dimensional contrastive loss synergy can dramatically boost the accuracy of RUL prediction, and the weight of timestamp continuity is prioritized over global discriminability in bearing degradation modeling.

**Table 12:** Impact of the hyper-parameter$\beta$on the model.

$\beta$	MSE	RMSE	MAE	${{\mathrm{R}}^2}$
0.1	0.0056	0.0750	0.0350	0.9218
0.2	0.0048	0.0695	0.0465	0.9328
**0.3**	**0.0036**	**0.0601**	**0.0297**	**0.9497**
0.4	0.0039	0.0627	0.0393	0.9454
0.5	0.0045	0.0670	0.0385	0.9376
0.6	0.0046	0.0680	0.0312	0.9358
0.7	0.0046	0.0675	0.0440	0.9367
0.8	0.0038	0.0618	0.0312	0.9468
0.9	0.0044	0.0664	0.0302	0.9388

## Conclusions

In this study, a novel bearing RUL prediction method based on a dual-dimensional contrastive SSL (DCSSL) was proposed. In the pretext task, the paradigm innovatively adopted the dilated causal convolution to model bearing degradation trends and strictly adhered to the temporal rules. Furthermore, it jointly utilized random cropping and timestamp masking techniques to construct positive pairs for contrastive learning, and optimized the contrastive learning loss simultaneously at the temporal-level and the instance-level of bearing degradation stages. In the downstream task, a newly created prediction head was fine-tuned to enable the model for RUL prediction. Validation on the FEMTO Bearing dataset demonstrated the method’s effectiveness in handling bearing degradation data with varying distributions and scales. Compared with existing advanced approaches, the proposed method exhibited superior performance across multiple evaluation metrics. Ablation studies confirmed the advantages of our designed model structure, the construction strategy of positive pairs, and the dual-dimensional contrastive learning framework. This provides a new solution for achieving precise RUL of rolling bearings.

This study and other state-of-the-art methods currently only considered situations without data distribution differences. However, in the RUL prediction across different operating conditions or devices, the changes in data distribution can significantly degrade model performance. Development of RUL prediction techniques based on transfer learning is future research.

This study proposed a novel method for bearing RUL prediction based on a dual-dimensional contrastive self-supervised learning (DCSSL) framework. In the pretext task, the model employed dilated causal convolution to capture the underlying degradation trends of bearings while rigorously preserving temporal dependencies. Positive pairs for contrastive learning were constructed by jointly applying random cropping and timestamp masking techniques. The learning process was optimized via a composite loss function that simultaneously operates at both the temporal and instance levels, corresponding to different stages of bearing degradation. For the downstream task, a newly designed prediction head was fine-tuned to adapt the model for accurate RUL prediction. Evaluations on the FEMTO Bearing dataset confirmed the method’s effectiveness in handling degradation data with varying distributions and scales. Compared with existing state-of-the-art approaches, the proposed method achieved superior performance across multiple evaluation metrics. Ablation studies further validated the advantages of the designed model architecture, the positive pair construction strategy, and the dual-dimensional contrastive learning framework. The study thus provides a promising solution for achieving precise RUL prediction of rolling bearings.

It should be noted that this study, like other current state-of-the-art methods, primarily addressed scenarios without significant cross-domain distribution shifts. In practical applications, however, variations in operating conditions or across different machinery can lead to substantial data distribution differences, which may considerably degrade model performance. Future research should, therefore, focus on developing RUL prediction techniques based on transfer learning to improve model generalizability under such distribution shifts.

## Data Availability

The FEMTO Bearing dataset used in this study are available at the following URL: https://phm-datasets.s3.amazonaws.com/NASA/10.+FEMTO+Bearing.zip.

## References

[1] Liu, Q. et al. A noval RUL prediction method for rolling bearing: TcLstmNet-CBAM. *Sci. Rep.* **15** (1), 14055. 10.1038/s41598-025-98845-9 (2025). 40269223 10.1038/s41598-025-98845-9 PMC12019386

[2] Ruan, D. et al. Light convolutional neural network by neural architecture search and model pruning for bearing fault diagnosis and remaining useful life prediction. *Sci. Rep.* **13** (1), 5484. 10.1038/s41598-023-31532-9 (2023). 37015955 10.1038/s41598-023-31532-9 PMC10073187

[3] Yin, Y., Tian, J. & Liu, X. Remaining useful life prediction based on parallel multi-scale feature fusion network. *J. Intell. Manuf.* **36** (5), 3111–3127. 10.1007/s10845-024-02399-y (2025).

[4] Qi, J., Mauricio, A. & Gryllias, K. Comparison of blind diagnostic indicators for condition monitoring of wind turbine gearbox bearings. *J. Eng. Gas Turbines Power*. **144** (4), 041019. 10.1115/1.4049797 (2022).

[5] Paris, P. & Erdogan, F. A critical analysis of crack propagation laws. *J. Basic Eng.* **85** (4), 528–533. 10.1115/1.3656900 (1963).

[6] Lai, J. J. et al. Weibull reliability regression model for prediction of bearing remaining useful life. *Comput. Aided Chem. Eng.* **49**, 829–834. 10.1016/B978-0-323-85159-6.50138-X (2022).

[7] Zhang, W. et al. A deep convolutional neural network with new training methods for bearing fault diagnosis under noisy environment and different working load. *Mech. Syst. Signal Process.* **100**, 439–453. 10.1016/j.ymssp.2017.06.022 (2018).

[8] Lin, H. et al. Matching pursuit network: an interpretable sparse time–frequency representation method toward mechanical fault diagnosis. *IEEE Trans. Neural Netw. Learn. Syst.*10.1109/TNNLS.2024.3483954 (2024). 39527434 10.1109/TNNLS.2024.3483954

[9] Zhao, Y. & Wang, Y. Remaining useful life prediction for multi-sensor systems using a novel end-to-end deep-learning method. *Measurement* **182**, 109685. 10.1016/j.measurement.2021.109685 (2021).

[10] Li, P. et al. An end-to-end neural network framework for state-of-health estimation and remaining useful life prediction of electric vehicle lithium batteries. *Renew. Sustain. Energy Rev.* **156**, 111843. 10.1016/j.rser.2021 (2022).

[11] Fu, S. et al. A novel time-series memory auto-encoder with sequentially updated reconstructions for remaining useful life prediction. *IEEE Trans. Neural Netw. Learn. Syst.* **33** (12), 7114–7125. 10.1109/TNNLS.2021 (2021). 10.1109/TNNLS.2021.3084249 34152990

[12] Shi, Z. & Chehade, A. A dual-LSTM framework combining change point detection and remaining useful life prediction. *Reliab. Eng. Syst. Saf.* **205**, 107257. 10.1016/j.ress.2020.107257 (2021).

[13] Guo, F., Niu, H. & Li, M. Remaining useful life prediction of precision bearing based on multi-head attention mechanism. *J. Phys.: Conf. Ser. ***2762**, 012053. 10.1088/1742-6596/2762/1/012053 (2024).

[14] Wei, H., Zhang, Q. & Gu, Y. Remaining useful life prediction of bearings based on self-attention mechanism, multi-scale dilated causal convolution, and temporal convolution network. *Meas. Sci. Technol.* **34** (4), 045107. 10.1088/1361-6501/acb0e9 (2023).

[15] Zhou, L. & Wang, H. MST-GAT: a multi-perspective spatial-temporal graph attention network for multi-sensor equipment remaining useful life prediction. *Inform. Fusion*. **110**, 102462. 10.1016/jinffus2024 (2024).

[16] Qin, Y. et al. Spatial-temporal multi-sensor information fusion network with prior knowledge embedding for equipment remaining useful life prediction. *Reliab. Eng. Syst. Saf.* **2025**, 111420. 10.1016/j.ress.2025.111420 (2025).

[17] Qi, J. et al. Attention-guided graph isomorphism learning: a multi-task framework for fault diagnosis and remaining useful life prediction. *Reliab. Eng. Syst. Saf.* **2025**, 111209. 10.1016/j.ress.2025.111209 (2025).

[18] Wang, L. et al. A gated graph convolutional network with multi-sensor signals for remaining useful life prediction. *Knowl. Based Syst.* **252**, 109340. 10.1016/j.knosys.2022.109340 (2022).

[19] Zhu, Q. et al. Contrastive BiLSTM-enabled health representation learning for remaining useful life prediction. *Reliab. Eng. Syst. Saf.* **249**, 110210. 10.1016/j.ress.2024.110210 (2024).

[20] Li, X., Ding, Q. & Sun, J. Q. Remaining useful life estimation in prognostics using deep Convolution neural networks. *Reliab. Eng. Syst. Saf.* **172**, 1–11. 10.1016/j.ress.2017.11.021 (2018).

[21] Su, X. et al. An end-to-end framework for remaining useful life prediction of rolling bearing based on feature pre-extraction mechanism and deep adaptive transformer model. *Comput. Ind. Eng.*, **161**, 107531. 10.1016/j.cie.2021.107531 (2021).

[22] An, X. et al. An enhanced approach for remaining useful life prediction of bearings using incomplete lifecycle data. *IEEE Access*10.1109/ACCESS.2025.3553279 (2025).

[23] Liu, H. et al. Remaining useful life prediction using a novel feature-attention-based end-to-end approach. *IEEE Trans. Industr. Inf.* **17** (2), 1197–1207. 10.1109/TII.2020.2983760 (2020).

[24] Jaiswal, A. et al. A survey on contrastive self-supervised learning. *Technologies* **9** (1), 2. 10.3390/technologies9010002 (2020).

[25] Wang, H. et al. Self-supervised signal representation learning for machinery fault diagnosis under limited annotation data. *Knowl. Based Syst.* **239**, 107978. 10.1016/j.knosys.2021.107978 (2022).

[26] Deng, W. et al. Bearings RUL prediction based on contrastive self-supervised learning. *IFAC-PapersOnLine* **56** (2), 11906–11911. 10.1016/j.ifacol.2023.10.604 (2023).

[27] Deng, Y. et al. Abnormal data detection for structural health monitoring: State-of-the-art review. *Dev. Built Environ.* **17**, 100337. 10.1016/j.dibe.2024.100337 (2024).

[28] An, Y. et al. Conditional self-supervised learning for few-shot classification. In *IJCAI* 2140–2146 (2021). 10.24963/ijcai.2021/295.

[29] Jing, L. & Tian, Y. Self-supervised visual feature learning with deep neural networks: a survey. *IEEE Trans. Pattern Anal. Mach. Intell.* **43** (11), 4037–4058. 10.1109/TPAMI.2020 (2020). 10.1109/TPAMI.2020.2992393 32386141

[30] Kong, Z. et al. A contrastive learning framework enhanced by unlabeled samples for remaining useful life prediction. *Reliab. Eng. Syst. Saf.* **234**, 109163. 10.1016/j.ress.2023.109163 (2023).

[31] He, K. et al. Momentum contrast for unsupervised visual representation learning. In *Proceedings of the IEEE/CVF Conference on Computer Vision and Pattern Recognition* 9729–9738 (2020). 10.1109/CVPR42600.2020.00975.

[32] Chen, T. et al. A simple framework for contrastive learning of visual representations. In *International Conference on Machine Learning* 1597–1607 (PmLR, 2020).

[33] Chen, P. et al. Progressive contrastive representation learning for defect diagnosis in aluminum disk substrates with a bio-inspired vision sensor. *Expert Syst. Appl.* **2025**, 128305. 10.1016/j.eswa.2025.128305 (2025).

[34] Oord, A. V. D., Li, Y. & Vinyals, O. Representation learning with contrastive predictive coding. *ArXiv Preprint* arXiv:1807.03748. 10.48550/arXiv.1807.03748 (2018).

[35] Wang, T. et al. Data-driven prognostic method based on self-supervised learning approaches for fault detection. *J. Intell. Manuf.* **31** (7), 1611–1619. 10.1007/s10845-018-1431-x (2020).

[36] Li, Y. et al. A self-supervised assisted label-efficient method for online remaining useful life prediction. *Measurement* **242**, 115902. 10.1016/j.measurement.2024.115902 (2025).

[37] Khosla, P. et al. Supervised contrastive learning. *Adv. Neural. Inf. Process. Syst.* **33**, 18661–18673. 10.48550/arXiv.2004.11362 (2020).

[38] Rajasegaran, J. et al. Self-supervised knowledge distillation for few-shot learning. arXiv preprint arXiv:2006.09785 (2020). 10.48550/arXiv.2006.09785.

[39] Van Den Oord, A. et al. Wavenet: a generative model for raw audio. *ArXiv Preprint arXiv:1609 03499*. 10.48550/arXiv.1609.03499 (2016).

[40] Franceschi, J. Y., Dieuleveut, A. & Jaggi, M. Unsupervised scalable representation learning for multivariate time series. *Adv. Neural. Inf. Process. Syst.* **32**, 2563. 10.48550/arXiv.1901.10738 (2019).

[41] Bai, S., Kolter, J. Z. & Koltun, V. An empirical evaluation of generic convolutional and recurrent networks for sequence modeling. *ArXiv Preprint* arXiv:1803.01271.10.48550/arXiv.1803.01271 (2018).

[42] Patrick, N. et al. PRONOSTIA: an experimental platform for bearings accelerated life test. In *IEEE International Conference on Prognostics and Health Management, Denver, CO, USA*. https://hal.science/hal-00719503 (2012).

[43] Luo, D. et al. Time series contrastive learning with information-aware augmentations. In *Proceedings of the AAAI Conference on Artificial Intelligence, vol. 37* 4534–4542. 10.1609/aaai.v37i4.25575 (2023).

[44] Zhang, K. et al. Self-supervised learning for time series analysis: taxonomy, progress, and prospects. *IEEE Trans. Pattern Anal. Mach. Intell.* **46** (10), 6775–6794. 10.1109/TPAMI.2024.3387317 (2024). 38598381 10.1109/TPAMI.2024.3387317

